# Malaria elimination on Hainan Island despite climate change

**DOI:** 10.1038/s43856-022-00073-z

**Published:** 2022-02-09

**Authors:** Huaiyu Tian, Naizhe Li, Yapin Li, Moritz U. G. Kraemer, Hua Tan, Yonghong Liu, Yidan Li, Ben Wang, Peiyi Wu, Bernard Cazelles, José Lourenço, Dongqi Gao, Dingwei Sun, Wenjing Song, Yuchun Li, Oliver G. Pybus, Guangze Wang, Christopher Dye

**Affiliations:** 1 State Key Laboratory of Remote Sensing Science, Center for Global Change and Public Health, College of Global Change and Earth System Science, Beijing Normal University, Beijing, China; 2 Central Theater Center for Disease Control and Prevention of PLA, Beijing, China; 3 Department of Zoology, University of Oxford, Oxford, UK; 4 School of Biomedical Informatics, the University of Texas Health Science Center at Houston, Houston, TX USA; 5 Institut de Biologie de l’École Normale Supérieure, Unité Mixte de Recherche, Centre National de la Recherche Scientifique et École Normale Supérieure, Paris, France; 6 Unité Mixte Internationnale, Mathematical and Computational Modeling of Complex Systems, Institut de Recherche pour le Développement et Sorbonne Université, Bondy, France; 7 Hainan Center for Disease Control and Prevention, Haikou, China; 8 Department of Pathobiology and Population Science, The Royal Veterinary College, London, UK; 9 Oxford Martin School, University of Oxford, Oxford, UK

**Keywords:** Infectious diseases, Diseases

## Abstract

**Background:**

Rigorous assessment of the effect of malaria control strategies on local malaria dynamics is a complex but vital step in informing future strategies to eliminate malaria. However, the interactions between climate forcing, mass drug administration, mosquito control and their effects on the incidence of malaria remain unclear.

**Methods:**

Here, we analyze the effects of interventions on the transmission dynamics of malaria (*Plasmodium vivax* and *Plasmodium falciparum*) on Hainan Island, China, controlling for environmental factors. Mathematical models were fitted to epidemiological data, including confirmed cases and population-wide blood examinations, collected between 1995 and 2010, a period when malaria control interventions were rolled out with positive outcomes.

**Results:**

Prior to the massive scale-up of interventions, malaria incidence shows both interannual variability and seasonality, as well as a strong correlation with climatic patterns linked to the El Nino Southern Oscillation. Based on our mechanistic model, we find that the reduction in malaria is likely due to the large scale rollout of insecticide-treated bed nets, which reduce the infections of *P. vivax* and *P. falciparum* malaria by 93.4% and 35.5%, respectively. Mass drug administration has a greater contribution in the control of *P. falciparum* (54.9%) than *P. vivax* (5.3%). In a comparison of interventions, indoor residual spraying makes a relatively minor contribution to malaria control (1.3%–9.6%).

**Conclusions:**

Although malaria transmission on Hainan Island has been exacerbated by El Nino Southern Oscillation, control methods have eliminated both *P. falciparum* and *P. vivax* malaria from this part of China.

## Plain language summary

Several malaria control strategies have been implemented on Hainan Island, China, and it is important to determine which of these have been effective to guide future efforts to control malaria. Here, we use mathematical and statistical methods to assess the effectiveness of control methods using data on malaria cases on Hainan, considering the impact of climate change simultaneously, since malaria transmission is affected by the climate. We observe time-related trends in malaria incidence and a strong relationship with climate before the large-scale rollout of malaria control interventions. We find that insecticide-treated bed nets are the most effective strategy in decreasing malaria incidence, while mass drug administration and indoor residual spraying also contribute to malaria control. Our findings provide evidence that a combination of strategies reduces the burden of malaria in affected regions.

## Abstract (web summary)

Tian et al. use mathematical modelling to estimate the impact of various interventions on malaria incidence on Hainan Island, also taking into account climate change. They find that although malaria transmission has been exacerbated by climate change, insecticide-treated bed nets and other interventions were effective in controlling the disease.

## Introduction

Malaria is a mosquito-borne infectious disease caused by single-celled microorganisms of the *Plasmodium* group and spread by *Anopheles* mosquitoes. Although global malaria incidence and mortality rates have decreased since 2000, it remains one of the world’s most serious public health concerns, with 228 million new cases reported in 2018^1^, particularly in lower and middle-income countries. Although the World Health Organization (WHO) has set the goal of reducing malaria burden by 90% by 2030^2^, a recent study has suggested that the complete eradication of malaria by 2050 may be feasible if the appropriate strategies and actions are implemented^3^. For endemic areas, the main priority is to control and ultimately eliminate malaria^4–6^. Considering many Southeast Asian countries are still trying to eliminate this disease by 2030 or 2040, there’s an urgent need to conduct studies using real-world data of malaria interventions in the west-pacific region.

A few countries have been successful in eliminating malaria using a combination of control strategies^4,7^ and those intervention methods have also been well documented^8–10^. In addition, changes in environmental conditions^11–18^ can drive malaria dynamics by influencing the mosquito and parasite life cycles^19–29^. Rainfall is required to establish suitable mosquito habitats, adequate levels of humidity enable high activity and survival of mosquitoes, and temperature affects multiple stages of mosquito and parasite development, as well as biting rates^30–32^. However, assessment of the effects of interventions on local malaria dynamics is complex^33–35^, and an evaluation of their effectiveness at reducing incidence is difficult to ascertain due to the presence of multiple factors^36,37^. Little is known about the effectiveness of interventions at reducing the malaria burden when accounting for the potential effects of climate changes^34,38^.

The epidemiological and parasitological surveys conducted on Hainan Island between 1995 and 2010 provide a longitudinal and comprehensive dataset for the assessment of the relative impacts of interventions, including mass antimalarial drug administration (MDA), indoor residual spraying (IRS), and insecticide-treated bed nets (ITNs), as well as climatic factors on driving malaria epidemics. Specifically, we quantify multiple exposures, nonlinear feedbacks, and complex interactions between interventions and transmission dynamics, which is an essential component of successful malaria control. A number of factors make the area well suited for analysis in this study; it has undergone both passive and active surveillance of cases, and our entomological survey indicates that mosquito population abundance has remained approximately constant over time^39,40^. In this study, we assess the effectiveness of control methods using mathematical and statistical methods. We find that ITNs are the most effective strategy in controlling malaria transmission in Hainan. It is also shown that MDA has a greater contribution in the control of *P. falciparum*.

## Methods

### Description of study sites

The study area Qiongzhong is located in the highland area of Hainan Island, China, with a population of more than 200,000. The county covers 2704.66 km^2^, and has a population density of over 70/km^2^. Its altitude ranges between 1200 m above sea level in the south to 1400 m in the north and to 1800 m in the west. The main epidemic season occurs from May to October during the rainy season.

### Epidemiological data

A time series of monthly cases of *Plasmodium falciparum* and *Plasmodium vivax* infections were obtained from the Hainan Provincial Centre for Disease Control and Prevention. Each case was confirmed through a microscopic examination of blood slides from clinical (febrile) patients seeking a diagnosis and treatment, according to the diagnostic criteria of the Chinese Ministry of Health^39^. Experimental procedures were performed in compliance with guidelines established by the Chinese Centre For Disease Control and Prevention and have been approved by ethics committee of Hainan Centre for Disease Control and Prevention. As the research involved no risk to the subjects and it used data from an anonymized dataset, informed consent was not required in this study.

Between 1995 and 2010, mass blood examinations were conducted annually, comprising 293,117 blood smears. Antimalarial drug campaigns were conducted biannually in both previously infected and uninfected people in April and August, together with vector control (indoor residual spraying, IRS) and prevention (insecticide-treated bed nets, ITNs). A total of 122,510 people took antimalarial medication, with a strategy that consume 8 days of piperaquine with primaquine. An area of 4,231,517 m^2^ was sprayed on indoor areas and 170,129 insecticide-treated nets were used, with the rate of bed net using ranging from 0.37 to 7.88 per 100 people each year. All these statistics were gathered in Qiongzhong highland. The time series for the population sizes of inhabitants was obtained from the Hainan Statistical Yearbook.

### Climate data

Climate data, including temperature and rainfall, were obtained from the local meteorological station in Qiongzhong from 1995 to 2010. The Niño 3.4 index^41^ is calculated as the difference between the monthly average sea surface temperatures in the region 5°N–5°S, 120°W–170°W. Only Niño 3.4 index was included as a climatic component in both statistical model and mathematical model.

### Statistical model

The associations between climate, antimalarial drug campaigns, IRS, and ITNs with malaria incidence were assessed with a statistical model, Generalized Additive Model (GAM). We fitted separate negative binomial regression models to the monthly incidence of *P. vivax* and *P. falciparum*.

$${Y}_{t} \sim {{{{{\rm{NegBin}}}}}}({\mu }_{t},\theta )$$

1 $$\log ({\mu }_{t})=\alpha +{f}_{{{{{\rm{SEAS}}}}}}({{{{{\rm{Month}}}}}}_{t})+\beta {{{{{\rm{CLIM}}}}}}{}_{t}+\gamma {{{{{\rm{MDA}}}}}}_{t}+\varphi {{{{{\rm{IRS}}}}}}_{t}+\lambda {{{{{\rm{INTs}}}}}}_{t}$$

where *Y* is the monthly malaria incidence, *t* is the time, *α* is the intercept, and *β*, *γ*, *φ* and *λ* are regression coefficients. *f*_SEAS_ is a nonlinear smoothed functions of seasonality. CLIM is Niño 3.4 index. IRS is the area of IRS and ITNs is the number of insecticide-treated bed nets. The statistical model was used for exploring initial relationships between climate, intervention factors, and malaria incidence. Only significant variables were included in the epidemic model.

### Epidemic modelling

To investigate the impact of climate condition and specific control intervention on malaria mitigation, we established a mechanistic model^20,42,43^ by fitting to the number of new confirmed cases reported each month using Bayesian Markov Chain Monte Carlo methods^44^. Comparing to these previous models, we included new parameters representing for ITNs and IRS in mosquito classes. To estimate the impact of MDA, the class T (antimalarial drug treatment with temporary immunity) has also been included. We used the fitted model, with posterior estimates of parameters (*SI Appendix*, Fig. S7, Table S2), to simulate malaria epidemic, with and without control measures. The model is:

*P. falciparum* model

$${{{{{\rm{d}}}}}}S1/{{{{{\rm{d}}}}}}t=bN-\lambda S1+{\mu }_{S2S1}S2+{\mu }_{TS1}T-{T}_{A}-{{{{{\rm{d}}}}}}S1$$

$${{{{{\rm{d}}}}}}E/{{{{{\rm{d}}}}}}t=\lambda S1-{\mu }_{EI}E-{{{{{\rm{d}}}}}}E$$

2 $${{{{{\rm{d}}}}}}I/{{{{{\rm{d}}}}}}t={\mu }_{EI}E-(1-{t}_{S}){\mu }_{IS2}I+{s}_{I}\lambda S2-{t}_{s}{\mu }_{IT}I-{{{{{\rm{d}}}}}}I$$

$${{{{{\rm{d}}}}}}S2/{{{{{\rm{d}}}}}}t=(1-{t}_{S}){\mu }_{IS2}I-{\mu }_{S2S1}S2-{s}_{I}\lambda S2-{T}_{T}-{{{{{\rm{d}}}}}}S2$$

$${{{{{\rm{d}}}}}}T/{{{{{\rm{d}}}}}}t={T}_{A}+{T}_{T}+{t}_{S}{\mu }_{IT}-{\mu }_{TS1}T-{{{{{\rm{d}}}}}}T$$

*P. vivax* model

$${{{{{\rm{d}}}}}}S1/{{{{{\rm{d}}}}}}t=bN-\lambda S1+{\mu }_{S2S1}S2+{\mu }_{TS1}T-{T}_{A}-{{{{{\rm{d}}}}}}S1$$

$${{{{{\rm{d}}}}}}E/{{{{{\rm{d}}}}}}t=\lambda S1-{\mu }_{EI}E-{{{{{\rm{d}}}}}}E$$

$${{{{{\rm{d}}}}}}I/{{{{{\rm{d}}}}}}t={\mu }_{EI}E-(1-{t}_{S}){\mu }_{IS2}{r}_{IH}I+{s}_{I}\lambda S2-{t}_{s}{\mu }_{IT}I+n{\mu }_{HI}-{{{{{\rm{d}}}}}}I$$

3 $${{{{{\rm{d}}}}}}{H}_{1}/{{{{{\rm{d}}}}}}t=(1-{t}_{S}){\mu }_{IS2}{r}_{IH}I-n{\mu }_{HI}{H}_{1}-{{{{{\rm{d}}}}}}{H}_{1}$$

$${{{{{\rm{d}}}}}}{H}_{i}/{{{{{\rm{d}}}}}}t=n{\mu }_{HI}{H}_{i-1}-n{\mu }_{HI}{H}_{i}-{{{{{\rm{d}}}}}}{H}_{i}\,[{{{{{\rm{for}}}}}}\,i=2,\ldots ,n]$$

$${{{{{\rm{d}}}}}}S2/{{{{{\rm{d}}}}}}t=(1-{t}_{S})(1-{r}_{IH}){\mu }_{IS2}I-{\mu }_{S2S1}S2-{s}_{I}\lambda S2-{T}_{T}-{{{{{\rm{d}}}}}}S2$$

$${{{{{\rm{d}}}}}}T/{{{{{\rm{d}}}}}}t={T}_{A}+{T}_{T}+{t}_{S}{\mu }_{IT}-{\mu }_{TS1}T-{{{{{\rm{d}}}}}}T$$

where human classes consist of *S*1 (susceptible), *E* (exposed), *I* (infected), *S*2 (recovered subpatent status, with partial immunity to reinfection), *H* (dormant liver stage), and *T* (antimalarial drug treatment with temporary immunity). Multiple *H*_*i*_ classes were used to partition the dormant stage into a sequence of identical stages (*i* = 2, …, *n*)^42^. *T*_*A*_ and *T*_*T*_ represent preventative antimalarial drug treatments administered to the general population and patients with previous malarial infections before the epidemic season in April and August every year, respectively. *S*1 + *E* + *I* + *H* + *S*2 + *T* = *N*, and *b* and *d* are birth and death rates of the population, both of which were obtained from statistical yearbook. *λ* is the force of infection at the current time. *μ* represents transition rate between classes. *t*_*s*_ is the treatment success probability. *s*_*I*_ represents superinfection from *S*2 to *I* and *r*_*IH*_ is the rate at which the infected population transits into dormant liver stage. The observed monthly cases equal the number of new cases predicted by the model multiplied by the reporting rate *r*. The annual number of reported cases is strongly correlated with positive rate of blood examinations in our study area, a constant reporting rate over time is then assumed.

The role of mosquitoes in transmission is represented through a delayed equation between the latent *f* and current *λ* force of infection, taking into account the extrinsic incubation period *τ*^20^.

$${{{{{\rm{d}}}}}}{f}_{1}/{{{{{\rm{d}}}}}}t=(f-{f}_{1})\kappa /\tau$$

4 $${{{{{\rm{d}}}}}}{f}_{i}/{{{{{\rm{d}}}}}}t=({f}_{i-1}-{f}_{i})\kappa /\tau$$

$${{{{{\rm{d}}}}}}\lambda /{{{{{\rm{d}}}}}}t=({f}_{\kappa -1}-\lambda )\kappa /\tau$$

5 $$f(t)=\frac{I(t)+{s}_{f}S2(t)}{N(t)}\beta (t)$$

6 $$\beta (t)={\beta }_{{{{{\rm{seas}}}}}}(t){\beta }_{{{{{\rm{trend}}}}}}(t)$$

7 $${\beta }_{{{{{\rm{trend}}}}}}(t)={{{{{\rm{CLIM}}}}}}(t)+{{{{{\rm{IRS}}}}}}(t)+{{{{{\rm{ITNs}}}}}}(t)$$

8 $${\beta }_{{{{{\rm{seas}}}}}}(t)=\mathop{\sum }\limits_{i=1}^{n}{\varphi }_{i}{\Delta }_{i}{{{{{\rm{Month}}}}}}_{i}$$

where IRS (*t*) = *θ*_IRS_ × area of IRS (m^2^/per person) (*t*), ITNs (*t*) = *θ*_ITN_ × coverage of insecticide-treated nets (*t*), CLIM (*t*) = *θ*_CLIM_ × Niño 3.4. Seasonality, *β*_seas_, is modelled nonparametrically through the coefficients *β*_*i*_ of the periodic cubic B-spline basis *φ*_*i*_ (*t*). Δ is a vector of dummy variables of length 12. We developed the model shown in Fig. S1. We use *k* = 2 in the model.

We fit the malaria model using a Bayesian state space framework. Model fitting was performed using Metropolis–Hastings Markov chain Monte Carlo (MCMC) algorithm with the MATLAB (version R2016b) toolbox DRAM (Delayed Rejection Adaptive Metropolis). In model parameterization, we chose a Gaussian prior for the distribution of parameters, with a mean value of 0 and a variance of 10^2^. After a burn-in of 1 million iterations, we ran the chain for 10 million iterations sampled at every 1000th step to avoid auto-correlation (*SI Appendix*, Fig. S7). We also fitted our model and estimated the parameters using 1995–2004 data, and then predicted malaria cases in 2005–2010. We found that our model is able to predict and capture the trend of malaria, which reflects the effect of interventions on malaria transmission (*SI Appendix*, Fig. S8).

### Wavelet

Wavelet analyses were performed to investigate periodicity of ecological time series^45–47^. Wavelet analysis estimates the spectral characteristics of time series as function of time^45,46^. This method with the wavelet power spectrum (WPS), allows us to determine the contribution of variance at different times and periods. Here for the wavelet decomposition we adopted Morlet wavelet to analyze the *P. vivax* and *P. falciparum* time series. Additionally, a bootstrapping method was used to evaluate the statistical significance of the WPS (more details are found in ref. ^47^).

### Reporting summary

Further information on research design is available in the Nature Research Reporting Summary linked to this article.

## Results

### Temporal trends

**Fig. 1:**
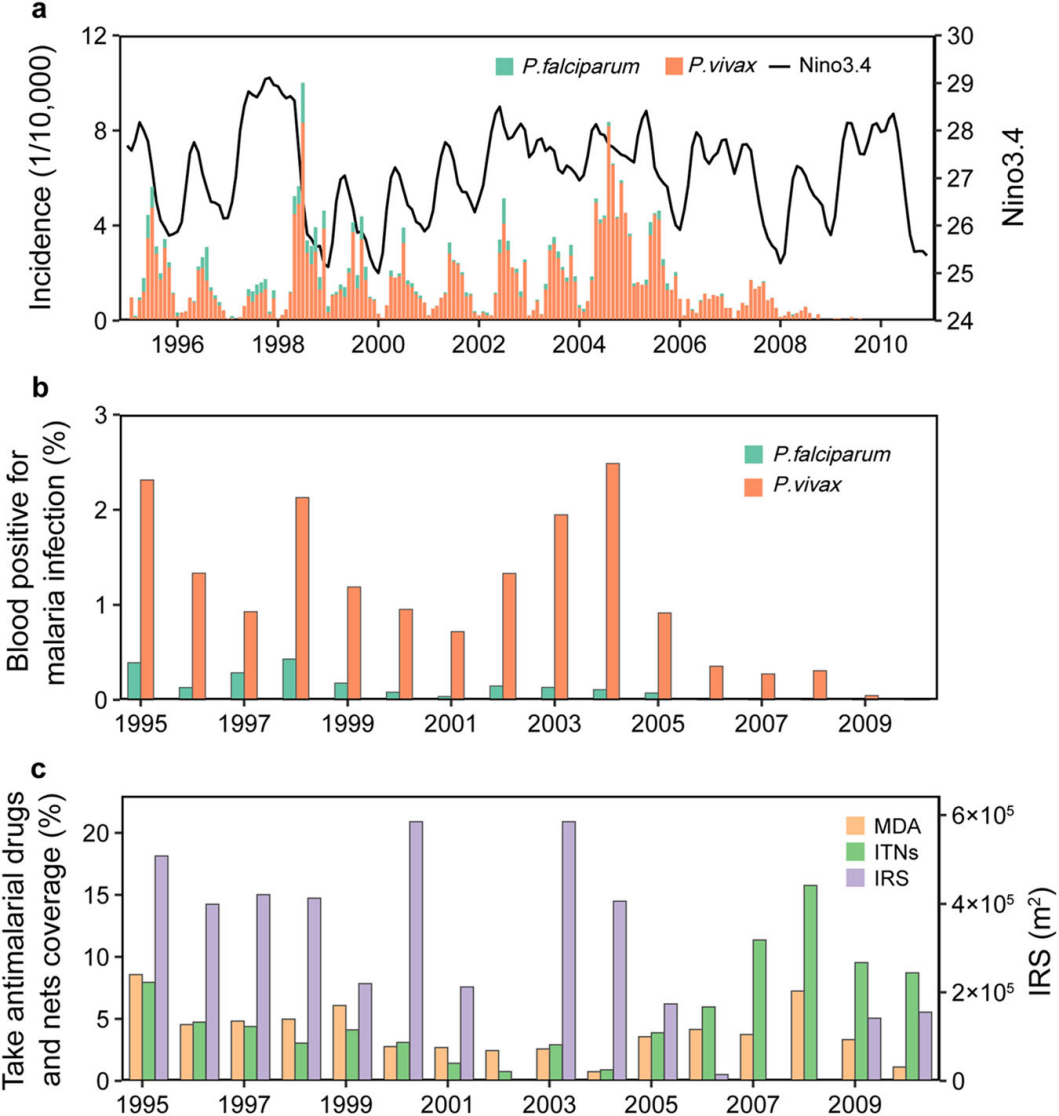
Summary of the Qiongzhong highland malaria epidemic between 1995 and the elimination of endemic malaria in 2010. **a** Monthly incidence of *Plasmodium vivax* (orange) and *Plasmodium falciparum* (green) and the Niño 3.4 index (black line). The Niño 3.4 index uses a 5-month running mean. **b** The changes in malaria prevalence in the population measured using an annual blood examination. **c** Malaria control strategy used in the study area. Antimalarial drug campaigns (orange), area of indoor spraying with the pesticide dicophane (DTT) (purple), and use of deltamethrin-treated nets (green). IRS, indoor residual spraying. ITNs, insecticide-treated bed nets. MDA, mass antimalarial drug administration.

Qiongzhong highland is located at the middle of Hainan Island, with a population of more than 200,000. The county covers 2704.66 km^2^ and has a population density of over 70/km^2^. Between 1995 and 2010, 5619 cases of malaria (598*P. falciparum* cases and 5021*P. vivax* cases) were reported in Qiongzhong, with an annual incidence ranging from 0.3 to 54.0 infections per 10,000 individuals (Fig. 1a) and a prevalence of 4.6–259.4 infections per 10,000 individuals (assessed by a blood examination of 293,117 individuals) (Fig. 1b). *Anopheles minimus* was the primary vector of malaria. All the above statistics were gathered in Qiongzhong highland.

Climate (particularly the Niño 3.4 index and sea surface temperature in the region 5°N–5°S, 120°W–170°W) is correlated with the long-term trends of malaria incidence. For example, a notable malaria outbreak occurred during the 1998 El Niño event (represented by the Niño 3.4 index). The incidence and prevalence of malaria increased with the Niño 3.4 index between 1999 and 2005, indicating a critical role of climate in malaria transmission, but decreased after 2006, which might due to the malaria control measures (Fig. 1a, b). Each year there was a steady increase in malaria incidence that began in February, reached a peak in the rainy season July, and then decreased (*SI Appendix*, Fig. S1). There was a 3-month time lag between peak malaria incidence and Niño 3.4 (*R* = 0.36, *P* < 0.01) and a 1-month between peak malaria incidence and temperature (*R* = 0.51, *P* < 0.01), suggesting that sea surface temperature-related changes potentially represent good indicators of the variability and magnitude of malaria epidemics. However, the peak in rainfall lagged behind the annual peak malaria incidence. Because rainfall is abundant in our study area (*SI Appendix*, Fig. S2), it will not be included in the further analyses reported in this study.

Meanwhile, we observe a dramatic decrease in malaria incidence and changes in endemic periodicity after 2005 (*SI Appendix*, Fig. S3), a period when large scale interventions were rolled out. This suggests that there may be an impact of control or prevention activities that have effectively reduced human-mosquito transmission (Fig. 1c).

### Transmission model and effects of malaria control interventions

**Fig. 2:**
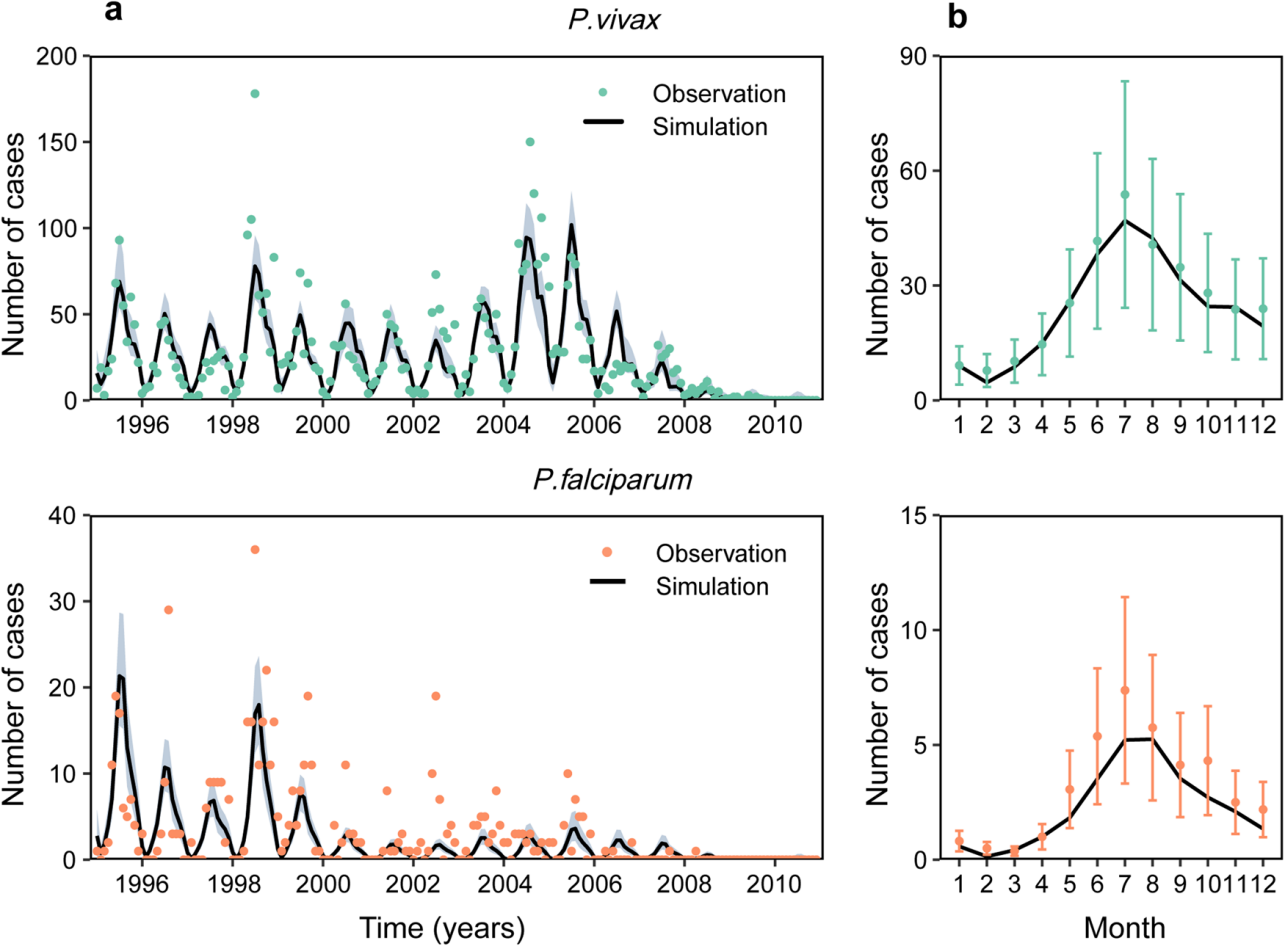
Mathematical model simulations of clinical malaria cases. **a** The time series of number of clinical cases. **b** The seasonal pattern of number of clinical cases. Observations are shown as points (number of reported malaria cases). The solid lines correspond to the simulations, and the dark grey indicates the 95% credible intervals. We only present the final results, focusing on the median of posterior distributions and 95% credible intervals extracted from 10,000 runs. Seasonal pattern for the observed cases (points and error bars represent average monthly cases and the 95% confidence interval, respectively) and the median of model simulations (black line).

**Fig. 3:**
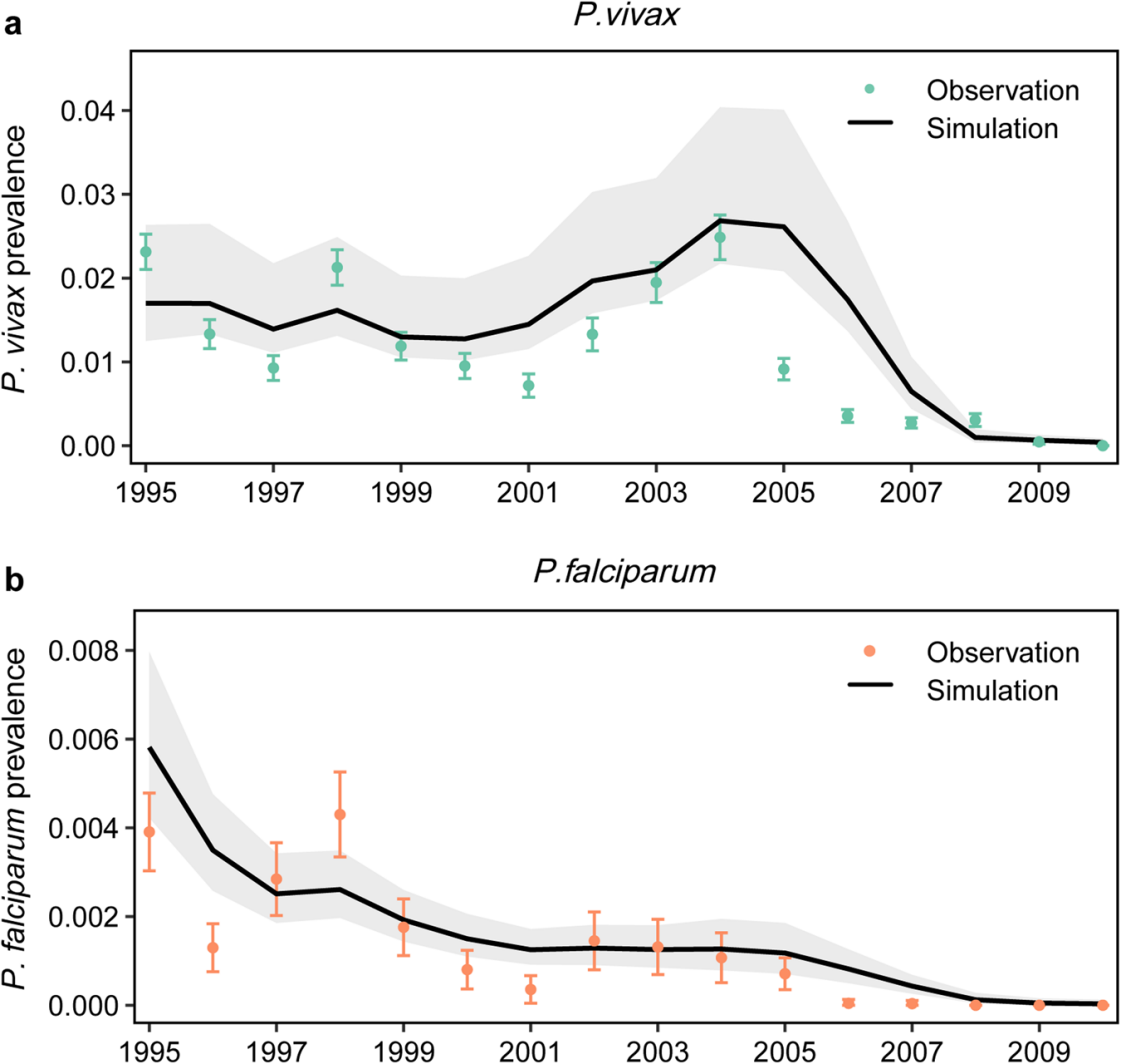
Malaria prevalence in humans. **a** *P. vivax* and **b** *P. falciparum*. The annual malaria prevalence was obtained from mass blood examinations. The observed data are plotted as green (*P. vivax*) and orange (*P. falciparum*) points with 95% confidence interval. The solid lines correspond to the malaria prevalence in humans estimated by epidemic model, and the grey areas correspond to pointwise 95% credible intervals.

To assess the effects of control strategies, we first used a negative binomial regression model to analyze how malaria incidence varied with climatic and intervention covariates (Table 1). The variables were in low collinearity with each other (*SI Appendix*, Table S1). The result of the regression model suggests that interventions were associated with a reduction in the number of cases. By fitting an epidemic model (*SI Appendix*, Fig. S4, Table S2) to the time series of reported cases, we investigated the possible effects of interventions on the trajectory of the epidemic. Transmission dynamics were modelled in five different ways: (i) only using a seasonal component (Seas), (ii) using a combination of Seas and the climatic component (CLIM), (iii) using a combination of Seas, CLIM, and MDA, (iv) using a combination of Seas, CLIM, MDA, and IRS, and (v) using a combination of Seas, CLIM, MDA, IRS, and insecticide-treated bed nets (ITNs). Our model (with ITNs) captures the observed seasonal/interannual patterns of the epidemics (Fig. 2) and is superior to models without ITNs (Table 2). In addition, the model also estimates the total number of *P. vivax* and *P. falciparum* infections (a constant reporting rate is assumed based on epidemiological investigation, *SI Appendix*, Fig. S5, Table S3), which exhibits a good fit to the annual malaria prevalence obtained from mass blood examinations over time (Fig. 3).

**Table 1:** Parameter estimates included in the negative binomial regression model.

Covariates	Coefficient (standard error)	
	*P. vivax*	*P. falciparum*
Intercept	−3.76 (2.27)	−5.10 (6.71)
Seasonality	EDF^a^ 3.75**	EFD 2.52
CLIM	0.17 (0.08)*	0.13 (0.24)
MDA	−0.09 (0.11)	0.09 (0.29)
IRS	0.11 (0.22)	−0.10 (0.64)
ITNs	−0.30 (0.04)**	−0.29 (0.12)*

*CLIM* climatic component, *MDA* mass antimalarial drug administration, *IRS* indoor residual spraying, *ITNs* insecticide-treated bed nets.

**P < 0.01, *P < 0.05.

^a^*EFD* effective degrees of freedom.

**Table 2:** Comparison of the dynamic models including the seasonal component, climatic component, vector control, and prevention of transmission of observed malaria epidemics.

Model	*P. vivax*		*P. falciparum*	
	*R*^2^	*DIC	*R*^2^	DIC
Seas	0.25	897.64	0.36	117.45
Seas + CLIM	0.34	893.27	0.47	117.72
Seas + CLIM + MDA	0.34	882.90	0.48	113.33
Seas + CLIM + MDA + IRS	0.33	832.35	0.48	114.88
Seas + CLIM + MDA + IRS + ITNs	0.68	500.14	0.49	109.44

*Seas* seasonal component, *CLIM* climatic component, *MDA* mass antimalarial drug administration, *IRS* indoor residual spraying, *ITNs* insecticide-treated bed nets. *DIC deviance information criterion.

**Fig. 4:**
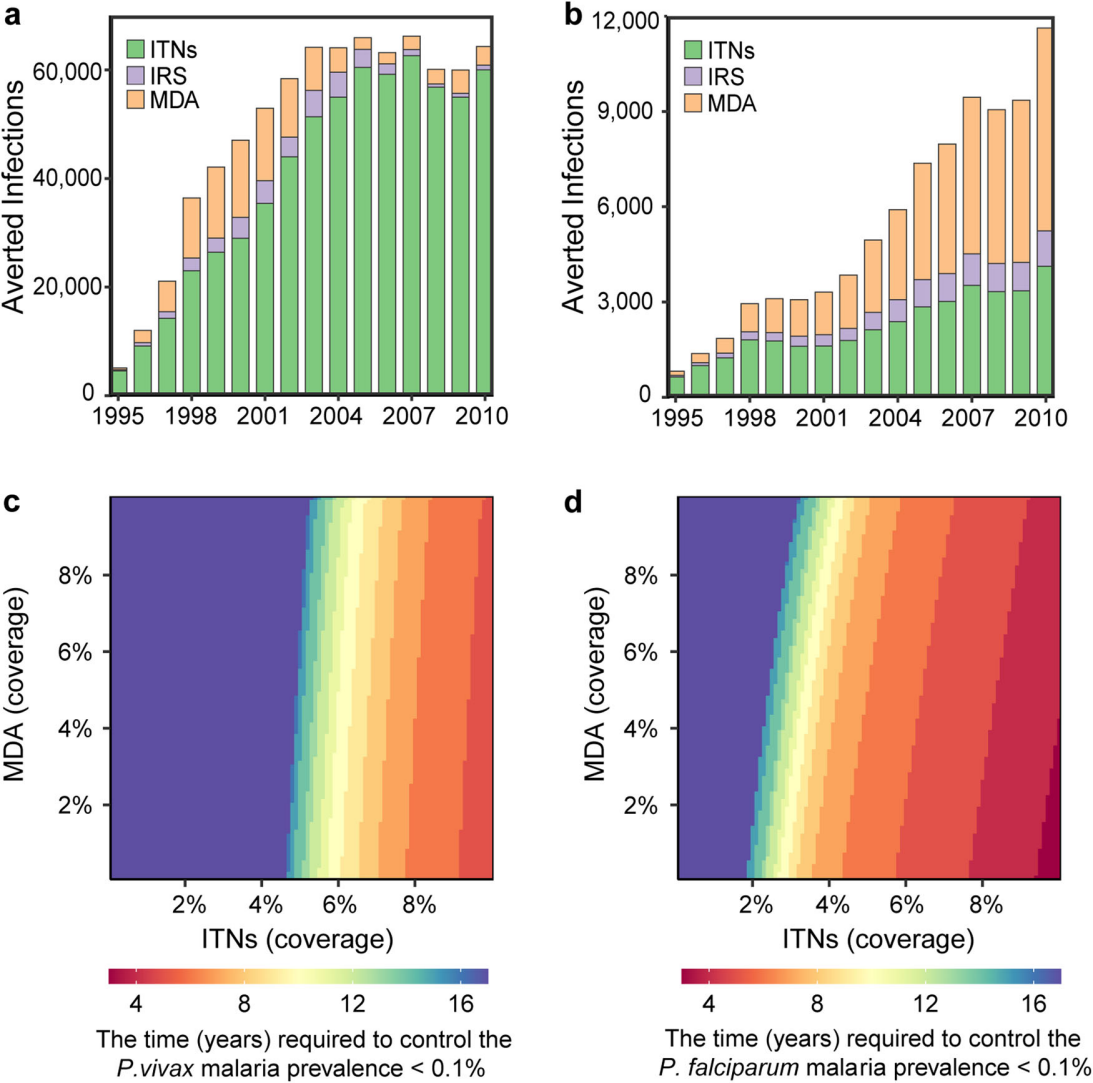
Changes in endemicity and effects of interventions conducted from 1995 to 2010. **a** *P. vivax* and **b** *P. falciparum*. Bars represent the predicted cumulative number of clinical malaria cases averted by interventions at the end of each year. The specific contribution of each intervention is distinguished after accounting for the effect of long-term climate forcing using mechanistic models. Mathematical models were fit to epidemiological data collected from 1995 to 2010, as well as ITNs, IRS, and MDA interventions. See the Methods for a detailed description of the mathematical models. **c** The time (years) required to control the annual malaria prevalence <0.1% for *P. vivax* and **d** *P. falciparum*. Malaria prevalence as a function of ITNs and MDA interventions in the simulation beginning in 1995. Estimates were obtained with all other covariates set to original values, such as climate conditions and population.

On the basis of the fit of the model to case reports, we investigated the possible effects of malaria control measures on the trajectory of the epidemic. Our model suggests that changes in the numbers of clinical and laboratory diagnosed cases of both *P. vivax* and *P. falciparum* after 2005 largely followed patterns of increased ITN coverage. Crucially, ITNs account for an estimated 93.4 and 35.5% of the decreases in the total infections of *P. vivax* and *P. falciparum* malaria by 2010, respectively (Fig. 4a, b). We estimate that malaria control interventions have prevented more than 7000 clinical cases since 1995 and that malaria prevalence decreased from 2.5% and 0.4% to less than 0.1% for *P. falciparum* and *P. vivax*, respectively, after the coverage of ITNs exceeded 15% in 2008 (Fig. 4a, b, *SI Appendix*, Fig. S6). According to our estimates, MDA had a greater contribution to eliminating *P. falciparum* malaria (54.9%) than *P. vivax* malaria (5.3%). Simulations highlight that increased use of ITNs and maintaining existing MDA would have reduced the malaria prevalence to less than 0.1% in a shorter period by reducing the transmission rate. Otherwise, this reduction in the regional infection and prevalence rates would have taken longer (Fig. 4c, d).

## Discussion

Using a process-based transmission model, we formulate a dynamic model of surveillance data that enables us to evaluate the effectiveness of interventions on reducing malaria incidence while simultaneously considering the impacts of climate change. We find that climate variability and intervention campaigns over the past few decades have shaped the pattern and long-term trend of malaria transmission in the highland area on Hainan Island. In Hainan, malaria has been successfully locally controlled, through a combination of control interventions, mainly due to the increased usage of ITNs on a large scale.

Previous studies have debated the relationship between climate factors and malaria transmission in China. Some studies reported a significant association between malaria transmission and temperature^48,49^ but others did not^50,51^. The effects of rainfall and humidity have also been debated^39,49^. In this study, we use Niño 3.4 index as the climate variable. Niño 3.4 index is one of several El Niño/ Southern Oscillation (ENSO) indicators based on sea surface temperature and could be used to reflect characters of temperature and rainfall. We found a significant association between Niño 3.4 and malaria transmission, which is consistent with previous studies^52,53^. The 3-month time lag between peak malaria incidence and Niño 3.4 indicates ENSO state could be used to predict malaria and including only one climate variable in the model has the benefit of reducing the impact of co-linearity. However, considering the so-called “ENSO spring barrier”, which means ENSO forecasts are intrinsically more uncertain connection with Northern Hemisphere spring, further researches should consider multiple study areas and various time units to evaluate the performance of ENSO forecasts on malaria prediction.

Our results are consistent with findings concerning the use of MDA to treat *P. falciparum*^8,54–56^, confirming that MDA reduces *P. falciparum* malaria transmission. However, our analysis further reveals a comparatively lower level of effectiveness of MDA (using chloroquine & primaquine) at eliminating *P. vivax* malaria, probably due to its ability to relapse from the dormant liver stage from weeks to years after clearance of the blood stage, which might allow this pathogen to maintain transmission during MDA treatment^33,57,58^. As total malaria incidence decreases, the proportion of infections due to *P. vivax* increases in Qiongzhong, Hainan Island. However, it’s difficult to precisely quantify the effectiveness of MDA because the evidence of patient compliance is not available. Furthermore, the increasing proportion of *P. vivax* malaria might result from the rapid decrease of *P. falciparum* malaria. In contrast to most previous studies, we provide a rare assessment of the differences in climate variability over time through transmission forcing and simultaneously compare the effectiveness of interventions on eliminating malaria risk using a mathematical model.

China has made great efforts to eliminate malaria, including strengthening the case reporting system, improving access to treatment, control of vectors, and health education^59^. The number of reported cases decreased dramatically with great changes  to epidemiological characteristics, which has benefited from powerful control methods^59–61^. Apart from the interventions included in our model, several other interventions have been proved useful in malaria control. For example, it is reported that behavioural change communication strategy significantly decreases malaria infections in mountain workers in Hainan province^62^. Improving knowledge on malaria is beneficial for the effectiveness of malaria-related interventions. It is also well known that socioeconomic development can reduce malaria risk and China has undergone substantial socioeconomic growth and urbanization since the 1980s^63,64^. These factors have also attributed to the decrease in malaria transmission in Qiongzhong highland. It may explain the declines in cases before 2005, when large scale interventions were rolled out, especially for *P. falciparum* malaria. The impact of these factors are not included in our analysis due to lacking of detailed records in such a long period.

Malaria control using a combination of ITNs and IRS has been shown to reduce the risk of malaria infection^65–69^. By incorporating field data and epidemiological records, our model explores the effects of multiple malaria control interventions used in combination, and ITNs might play the main role in eliminating malaria. Although confounding effects cannot be excluded (such as the socio-economic growth and housing improvement are associated with the decline of malaria transmission), ITNs appear to reduce the risk of malaria infection in Qiongzhong, including both the malaria incidence and prevalence, particularly for *P. vivax* malaria, since the coverage of ITNs has increased more than two- to three-fold to nearly 16%. However, due to the lack of experimental studies, we are unable to assess the effectiveness of a single intervention alone in the present study. Encouragingly, if high ITN coverage is achieved, malaria transmission can be controlled locally, even when climate conditions are favourable for high intensity transmission.

One limitation of our study is that the results must be interpreted with caution due to data availability. The lack of data about other factors, such as socioeconomic changes and urbanization, might induce an overestimation of the effect of interventions included in our model. We use the people who took antimalarial medication and number of insecticide-treated nets, but data about their compliance and the proportion of people sleeping under bed nets are not available. It hinders us to understand the relationship between interventions and malaria transmission more accurately. Similarly, insecticide resistance was not considered due to data availability. Although insecticide resistance might have reduced the effect of the massive scale-up of insecticide-treated bed nets because they would be less effective at killing mosquitoes, a multicountry cohort study indicates that insecticide-treated bed nets provide some protection against malaria, regardless of the presence of insecticide resistance^36^. Besides, our model was developed for *P. falciparum* and *P. vivax* separately. Although a higher risk of *P. vivax* malaria after treatment for *P. falciparum* infection in co-endemic regions has been described in a previous study from Thailand^70,71^, other factors, such as economic development, urbanization, and improvement in living conditions, and even human behaviour, change over time and might contribute to an overall reduction in the infection risk^63^. However, in the first 10 years, the epidemic magnitude (*P. falciparum* and *P. vivax*) remains constant until the massive scale-up of insecticide-treated bed net use. Furthermore, mosquito ecology was not included in our model, which is complex and changes non-linearly along with climate change and human behaviour especially the use of interventions such as IRS and ITNs^72,73^. Fortunately, only two species, *Anopheles minimus* and *Anopheles dirus*, were verified as major malaria vectors on Hainan island, which might reduce the uncertainties of our estimation. Our routine monitoring and previous studies^74,75^ show that these two species of mosquitoes still prefer to bite at night, when people spend more time at home and sleeping.

In summary, this study provides evidence that treating malaria with a combination of strategies controls malaria transmission and reduces the burden of malaria in co-endemic regions. Additionally, our findings are generalizable to regions in Southeast Asia, as our intervention provides easy access to health services. Our results comprise one component of a broad evidence baseline, including the effectiveness of malaria interventions and their interactions with climate variability, which should be considered in disease burden calculations and future risk models. It is important to apply our model to wider epidemiological zones.

## Supplementary information

Reporting Summary

Supplementary information

## Data Availability

The source data for the main figures in the manuscript can be available in GitHub, https://github.com/huaiyutian/Malaria-dynamic-model-Hainan-Qiongzhong and Zenodo, 10.5281/zenodo.5815265^76^. The data of population size can be obtained from Hainan Provincial Bureau of Statistics, http://stats.hainan.gov.cn/tjj/. The Niño 3.4 index can be obtained from the NOAA Physical Sciences Laboratory (PSL), https://www.psl.noaa.gov/. The malaria data that support the findings of this study are available from Hainan Centre for Disease Control and Prevention but restrictions apply to the availability of these data, which were used under license for the current study, and so are not publicly available. Data are however available from the authors upon reasonable request and with permission of Hainan Centre for Disease Control and Prevention. The climate data are available from National Meteorological Information Centre but restrictions apply to the availability of these data, which were used under license for the current study, and so are not publicly available. Data are however available from the authors upon reasonable request and with permission of National Meteorological Information Centre.

## Code availability

The code used in this study is available on GitHub, https://github.com/huaiyutian/Malaria-dynamic-model-Hainan-Qiongzhong and Zenodo, 10.5281/zenodo.5815265^76^.

## References

[1] World Health Organization. *World Malaria Report 2019* (World Health Organization, 2019).

[2] WHO Global Malaria Programme. *Global Technical Strategy for Malaria 2016–2030* (World Health Organization, 2015).

[3] Ghebreyesus TA (2019). The malaria eradication challenge. Lancet.

[4] Rabinovich RN (2017). malERA: An updated research agenda for malaria elimination and eradication. PLoS Med..

[5] Tatem AJ (2010). Ranking of elimination feasibility between malaria-endemic countries. Lancet.

[6] Chiyaka C (2013). The stability of malaria elimination. Science.

[7] World Health Organization. *Eliminating Malaria* (WHO, 2016).

[8] Brady OJ (2017). Role of mass drug administration in elimination of Plasmodium falciparum malaria: a consensus modelling study. Lancet Glob. Health.

[9] Tizifa TA (2018). Prevention efforts for malaria. Curr. Trop. Med. Rep..

[10] Laneri K (2015). Dynamical malaria models reveal how immunity buffers effect of climate variability. Proc. Natl Acad. Sci. USA.

[11] Ferguson NM (2018). Challenges and opportunities in controlling mosquito-borne infections. Nature.

[12] Siraj A (2014). Altitudinal changes in malaria incidence in highlands of Ethiopia and Colombia. Science.

[13] Lindblade KA, Walker ED, Onapa AW, Katungu J, Wilson ML (2000). Land use change alters malaria transmission parameters by modifying temperature in a highland area of Uganda. Trop. Med. Int. Health.

[14] Shanks GD, Hay SI, Omumbo JA, Snow RW (2005). Malaria in Kenya’s western highlands. Emerg. Infect. Dis..

[15] Hay SI (2002). Climate change and the resurgence of malaria in the East African highlands. Nature.

[16] Pascual M, Ahumada JA, Chaves LF, Rodo X, Bouma M (2006). Malaria resurgence in the East African highlands: Temperature trends revisited. Proc. Natl Acad. Sci. USA.

[17] Baeza A, Santos-Vega M, Dobson AP, Pascual M (2017). The rise and fall of malaria under land-use change in frontier regions. Nat. Ecol. Evol..

[18] Paaijmans KP, Read AF, Thomas MB (2009). Understanding the link between malaria risk and climate. Proc. Natl Acad. Sci. USA.

[19] Parham PE, Michael E (2009). Modeling the effects of weather and climate change on malaria transmission. Environ. Health Perspect..

[20] Laneri K (2010). Forcing versus feedback: Epidemic malaria and monsoon rains in northwest India. PLoS Comput. Biol..

[21] Bayoh, M. N. *Studies on the Development and Survival of Anopheles Gambiae Sensu Stricto at Various Temperatures and Relative Humidities.* PhD thesis, Durham University (2001).

[22] Boyd MF, Stratman-Thomas WK (1933). A note on the transmission of quartan malaria by *Anopheles Quadrimaculatus*. Am. J. Trop. Med. Hyg..

[23] Knowles R, Basu B (1943). Laboratory studies on the infectivity of *Anopheles stephensi*. J. Mal. Inst. India.

[24] Siddons L (1944). Observations on the influence of atmospheric temperature and humidity on the infectivity of *Anopheles culicifacies Giles*. J. Mal. Inst. India.

[25] Shute P, Maryon M (1952). A study of human malaria oocysts as an aid to species diagnosis. Trans. R. Soc. Trop. Med. Hyg..

[26] Vaughan JA, Noden BH, Beier JC (1992). Population dynamics of *Plasmodium falciparum* sporogony in laboratory-infected *Anopheles gambiae*. J. Parasitol..

[27] Eling W, Hooghof J, van de Vegte-Bolmer M, Sauerwein R, Van Gemert G (2001). Tropical temperatures can inhibit development of the human malaria parasite Plasmodium falciparum in the mosquito. Proc. Exp. Appl. Entomol..

[28] Bayoh M, Lindsay S (2003). Effect of temperature on the development of the aquatic stages of *Anopheles gambiae* sensu stricto (Diptera: Culicidae). Bull. Entomol. Res..

[29] Delatte H, Gimonneau G, Triboire A, Fontenille D (2009). Influence of temperature on immature development, survival, longevity, fecundity, and gonotrophic cycles of Aedes albopictus, vector of chikungunya and dengue in the Indian Ocean. J. Med. Entomol..

[30] Lardeux FJ, Tejerina RH, Quispe V, Chavez TK (2008). A physiological time analysis of the duration of the gonotrophic cycle of *Anopheles pseudopunctipennis* and its implications for malaria transmission in Bolivia. Malar. J..

[31] Stratman-Thomas WK (1940). The influence of temperature on Plasmodium vivax. Am. J. Trop. Med. Hyg..

[32] Mordecai EA (2013). Optimal temperature for malaria transmission is dramatically lower than previously predicted. Ecol. Lett..

[33] White MT (2018). Mathematical modelling of the impact of expanding levels of malaria control interventions on *Plasmodium vivax*. Nat. Commun..

[34] Bhatt S (2015). The effect of malaria control on Plasmodium falciparum in Africa between 2000 and 2015. Nature.

[35] Gething PW (2010). Climate change and the global malaria recession. Nature.

[36] Kleinschmidt I (2018). Implications of insecticide resistance for malaria vector control with long-lasting insecticidal nets: A WHO-coordinated, prospective, international, observational cohort study. Lancet Infect. Dis..

[37] Parham PE, Hughes DA (2015). Climate influences on the cost-effectiveness of vector-based interventions against malaria in elimination scenarios. Philos. Trans. R. Soc. Lond. B Biol. Sci..

[38] Lim SS (2011). Net benefits: a multicountry analysis of observational data examining associations between insecticide-treated mosquito nets and health outcomes. PLoS Med..

[39] Xiao D (2012). Epidemic distribution and variation of Plasmodium falciparum and Plasmodium vivax malaria in Hainan, China during 1995–2008. Am. J. Trop. Med. Hyg..

[40] Wang SQ (2014). Prevention measures and socio-economic development result in a decrease in malaria in Hainan, China. Malar. J..

[41] Trenberth KE, Stepaniak DP (2001). Indices of el niño evolution. J. Clim..

[42] Roy M, Bouma MJ, Ionides EL, Dhiman RC, Pascual M (2013). The potential elimination of Plasmodium vivax malaria by relapse treatment: insights from a transmission model and surveillance data from NW India. PLoS Negl. Trop. Dis..

[43] Rodó X, Martinez PP, Siraj A, Pascual M (2021). Malaria trends in Ethiopian highlands track the 2000 ‘slowdown’in global warming. Nat. Commun..

[44] Morton A, Finkenstädt BF (2005). Discrete time modelling of disease incidence time series by using Markov chain Monte Carlo methods. J. R. Stat. Soc. C.

[45] Cazelles B (2008). Wavelet analysis of ecological time series. Oecologia.

[46] Cazelles B, Chavez M, de Magny GC, Guégan J-F, Hales S (2007). Time-dependent spectral analysis of epidemiological time-series with wavelets. J. R. Soc. Interface.

[47] Cazelles B, Cazelles K, Chavez M (2014). Wavelet analysis in ecology and epidemiology: Impact of statistical tests. J. R. Soc. Interface.

[48] Zhang Y, Bi P, Hiller JE (2010). Meteorological variables and malaria in a Chinese temperate city: A twenty-year time-series data analysis. Environ. Int..

[49] Bi P, Tong S, Donald K, Parton KA, Ni J (2003). Climatic variables and transmission of malaria: A 12-year data analysis in Shuchen County, China. Public Health Rep..

[50] Wen L (2008). Spatial epidemiological study on malaria epidemics in Hainan province. Zhonghua Liu Xing Bing Xue Za Zhi.

[51] Tian L (2008). One-year delayed effect of fog on malaria transmission: A time-series analysis in the rain forest area of Mengla County, south-west China. Malar. J..

[52] Mantilla G, Oliveros H, Barnston AG (2009). The role of ENSO in understanding changes in Colombia’s annual malaria burden by region, 1960–2006. Malar. J..

[53] Kovats RS, Bouma MJ, Hajat S, Worrall E, Haines A (2003). El Niño and health. Lancet.

[54] Landier J (2018). Effect of generalised access to early diagnosis and treatment and targeted mass drug administration on Plasmodium falciparum malaria in Eastern Myanmar: an observational study of a regional elimination programme. Lancet.

[55] Kyelem D (2008). Determinants of success in national programs to eliminate lymphatic filariasis: a perspective identifying essential elements and research needs. Am. J. Trop. Med. Hyg..

[56] White NJ (2017). Does antimalarial mass drug administration increase or decrease the risk of resistance?. Lancet Infect. Dis..

[57] White NJ (2011). Determinants of relapse periodicity in *Plasmodium vivax* malaria. Malar J.

[58] White MT, Shirreff G, Karl S, Ghani AC, Mueller I (2016). Variation in relapse frequency and the transmission potential of *Plasmodium vivax* malaria. Proc. Biol. Sci..

[59] Lai S (2019). Changing epidemiology and challenges of malaria in China towards elimination. Malar. J..

[60] Zhang Q (2014). The epidemiology of Plasmodium vivax and Plasmodium falciparum malaria in China, 2004–2012: From intensified control to elimination. Malar. J..

[61] Lai S (2017). Malaria in China, 2011–2015: An observational study. Bull. World Health Organ.

[62] He CH (2014). Eliminating Plasmodium falciparum in Hainan, China: A study on the use of behavioural change communication intervention to promote malaria prevention in mountain worker populations. Malar. J..

[63] Tusting LS (2013). Socioeconomic development as an intervention against malaria: A systematic review and meta-analysis. Lancet.

[64] Gaughan AE (2016). Spatiotemporal patterns of population in mainland China, 1990 to 2010. Sci. Data.

[65] Kleinschmidt I (2006). Reduction in infection with Plasmodium falciparum one year after the introduction of malaria control interventions on Bioko Island, Equatorial Guinea. Am. J. Trop. Med. Hyg..

[66] Hemingway J, Beaty BJ, Rowland M, Scott TW, Sharp BL (2006). The Innovative Vector Control Consortium: Improved control of mosquito-borne diseases. Trends Parasitol.

[67] Kleinschmidt I (2009). Combining indoor residual spraying and insecticide-treated net interventions. Am. J. Trop. Med. Hyg..

[68] Yukich JO (2008). Costs and consequences of large-scale vector control for malaria. Malar. J..

[69] Hancock PA (2009). Combining fungal biopesticides and insecticide-treated bednets to enhance malaria control. PLoS Comput. Biol..

[70] Douglas NM (2011). Plasmodium vivax recurrence following falciparum and mixed species malaria: Risk factors and effect of antimalarial kinetics. Clin. Infect. Dis..

[71] Commons RJ (2019). Risk of Plasmodium vivax parasitaemia after Plasmodium falciparum infection: A systematic review and meta-analysis. Lancet Infect. Dis..

[72] Spitzen J, Koelewijn T, Mukabana WR, Takken W (2017). Effect of insecticide-treated bed nets on house-entry by malaria mosquitoes: The flight response recorded in a semi-field study in Kenya. Acta Trop..

[73] Grieco JP, Achee NL, Andre RG, Roberts DR (2000). A comparison study of house entering and exiting behavior of Anopheles vestitipennis (Diptera: Culicidae) using experimental huts sprayed with DDT or deltamethrin in the southern district of Toledo, Belize, C.A. J. Vector Ecol..

[74] Wu KC (1993). Studies on distribution and behavior of Anopheles minimus and its role of malaria transmission in Hainan Province at present. Zhongguo Ji Sheng Chong Xue Yu Ji Sheng Chong Bing Za Zhi.

[75] Zeng LH (2015). Analysis of the surveillance data about malaria vector in Hainan from 2005 to 2014. China Trop. Med..

[76] Tian, H. Code for: Malaria-dynamic-model-Hainan-Qiongzhong: Malaria-Qiongzhong. Zenodo 10.5281/zenodo.5815265 (2022).

