# Author Correction: Malaria elimination on Hainan Island despite climate change

**DOI:** 10.1038/s43856-022-00089-5

**Published:** 2022-02-24

**Authors:** Huaiyu Tian, Naizhe Li, Yapin Li, Moritz U. G. Kraemer, Hua Tan, Yonghong Liu, Yidan Li, Ben Wang, Peiyi Wu, Bernard Cazelles, José Lourenço, Dongqi Gao, Dingwei Sun, Wenjing Song, Yuchun Li, Oliver G. Pybus, Guangze Wang, Christopher Dye

**Affiliations:** 1 State Key Laboratory of Remote Sensing Science, Center for Global Change and Public Health, College of Global Change and Earth System Science, Beijing Normal University, Beijing, China; 2 Central Theater Center for Disease Control and Prevention of PLA, Beijing, China; 3 Department of Zoology, University of Oxford, Oxford, UK; 4 School of Biomedical Informatics, the University of Texas Health Science Center at Houston, Houston, TX USA; 5 Institut de Biologie de l’École Normale Supérieure, Unité Mixte de Recherche, Centre National de la Recherche Scientifique et École Normale Supérieure, Paris, France; 6 Unité Mixte Internationnale, Mathematical and Computational Modeling of Complex Systems, Institut de Recherche pour le Développement et Sorbonne Université, Bondy, France; 7 Hainan Center for Disease Control and Prevention, Haikou, China; 8 Department of Pathobiology and Population Science, The Royal Veterinary College, London, UK; 9 Oxford Martin School, University of Oxford, Oxford, UK

**Keywords:** Infectious diseases, Diseases

Correction to: *Communications Medicine* 10.1038/s43856-022-00073-z, published online 9 February 2022.

In the Acknowledgements section of this article the grant number relating to National Natural Science Foundation of China was incorrectly given as 82073616 and should have been 81460520. The original article has been corrected.

