# Supplementary material for: Malaria elimination on Hainan Island despite climate change
Source: Commun Med (Lond). 2022 Feb 9;2:12. doi: 10.1038/s43856-022-00073-z (PMC9053252; doi:10.1038/s43856-022-00073-z)
Supplement: Supplementary file 2 — Supplementary information [file 43856_2022_73_MOESM2_ESM.pdf]

Supplementary Information for

Malaria elimination on Hainan Island despite climate change

Huaiyu Tian[1]*, Naizhe Li[1], Yapin Li[2], Moritz U.G. Kraemer[4,5,6], Hua Tan[7], Yonghong Liu[1], Yidan Li[1], Ben Wang[1], Peiyi Wu[1], Bernard Cazelles[8,9], José Lourenço[4], Dongqi Gao[2], Dingwei Sun[3], Wenjing Song[2], Yuchun Li[3], Oliver G. Pybus[4,10], Guangze Wang[3]*, Christopher Dye[4,11]*

Correspondence to:

Huaiyu Tian, tianhuaiyu@gmail.com

Christopher Dye, christopher.dye@zoo.ox.ac.uk

Guangze Wang, wangguangze63@126.com

†These authors contributed equally to this work.

This file includes:

Figs. S1 to S8

Tables S1 to S3

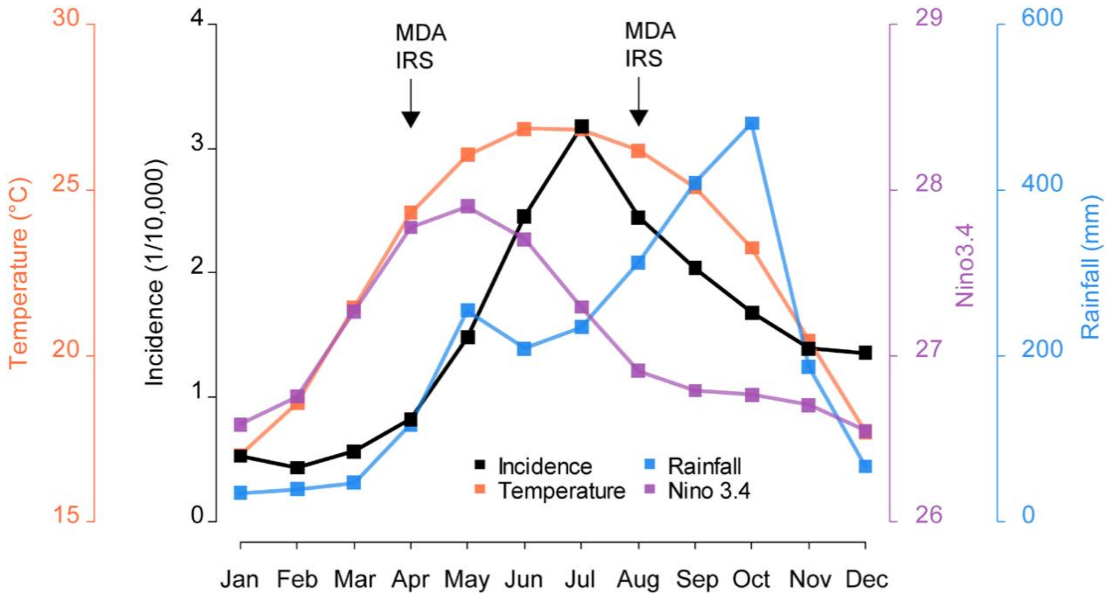

Fig. S1. Malaria transmission dynamics in Qiongzhong. Seasonality of the malaria incidence (black), temperature (orange), rainfall (blue), and Niño 3.4 index (purple) in Qiongzhong, 1995–2010. Mean monthly values are shown. MDA, mass antimalarial drug administration. IRS, indoor residual spraying. Arrows represent the biannual MDA and IRS.

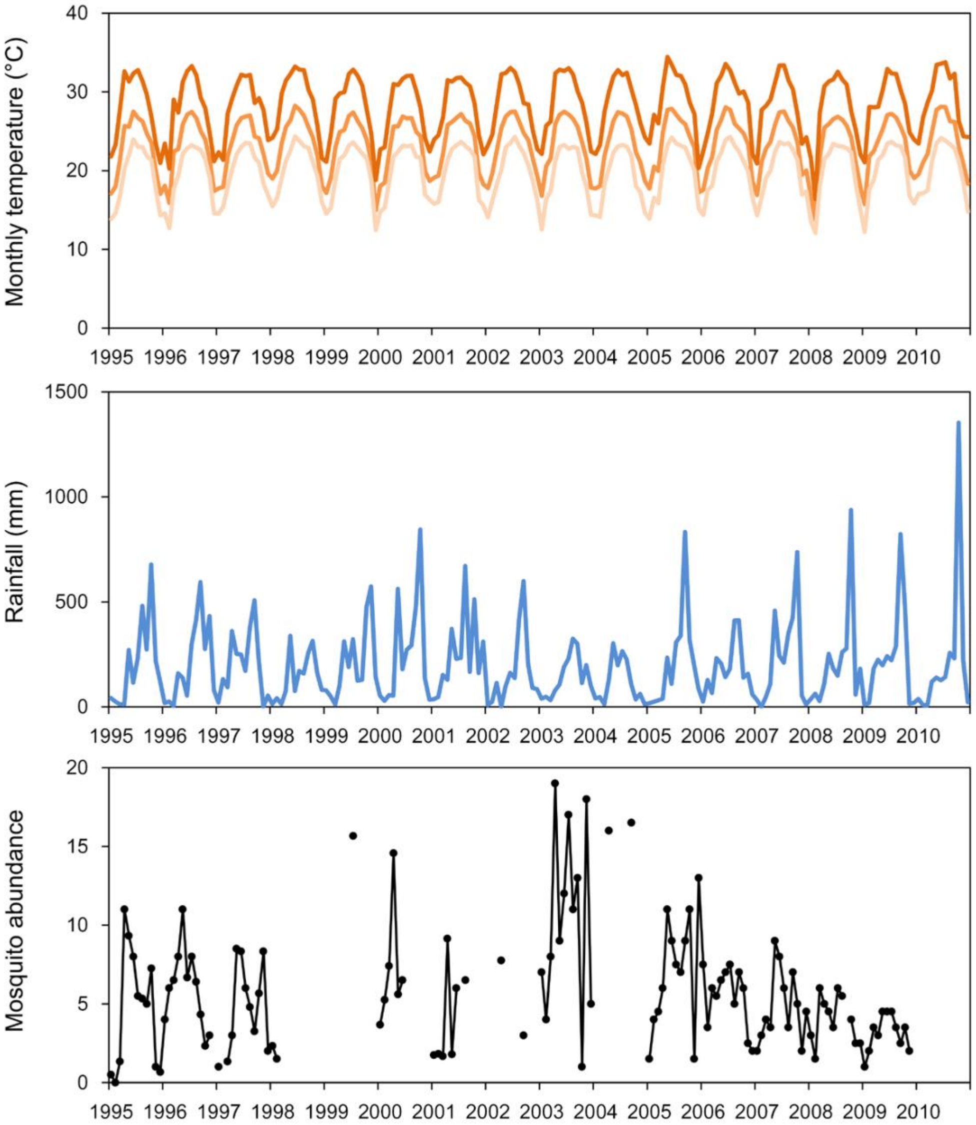

Fig. S2. Monthly temperature (orange), rainfall (blue), and mosquito abundance (black) in Qiongzhong, Hainan, China, from 1995-2010. For mosquito abundance, indoor and outdoor human landing catches were conducted monthly. *Anopheles minimus* was the primary vector of malaria in our study area. A total of 3889 mosquitoes were caught in 431 sites and constitute 148 time points (months).

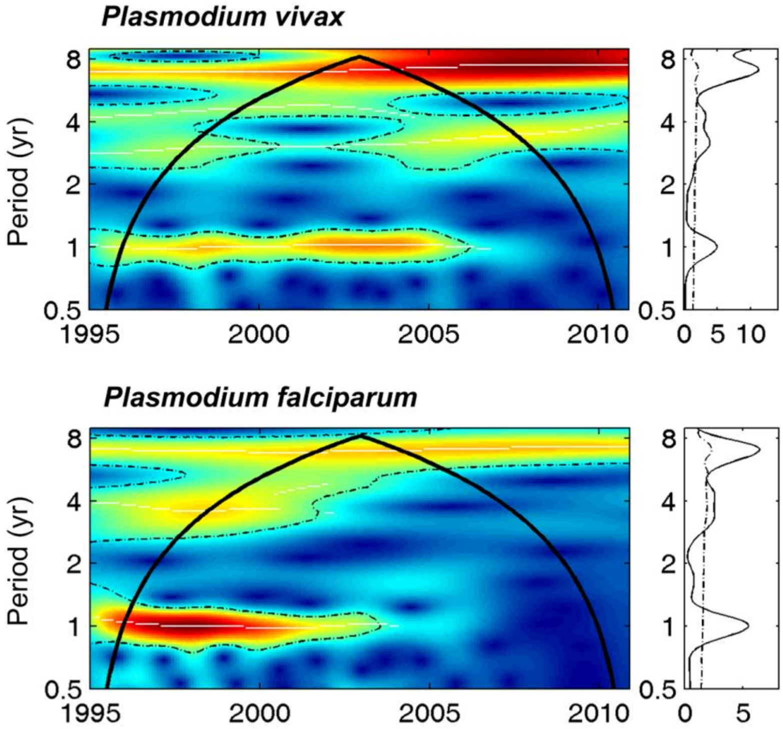

Fig. S3. Wavelet power spectra of the monthly time series showing the periodicity of the incidence of Plasmodium vivax (*upper panel*) and Plasmodium falciparum (*lower panel*). In the wavelet power spectra, the dotted-dashed lines represent statistical significance areas ($P < 0.05$) computed with 1000 bootstrapped time series, and the bold line is known as the cone of influence. Colors indicate the power of the wavelet, where red to blue represent strong to weak power and the white line indicates the maximum power. Right shows the global wavelet spectra with its significant threshold value of 5% (dotted-dashed line).

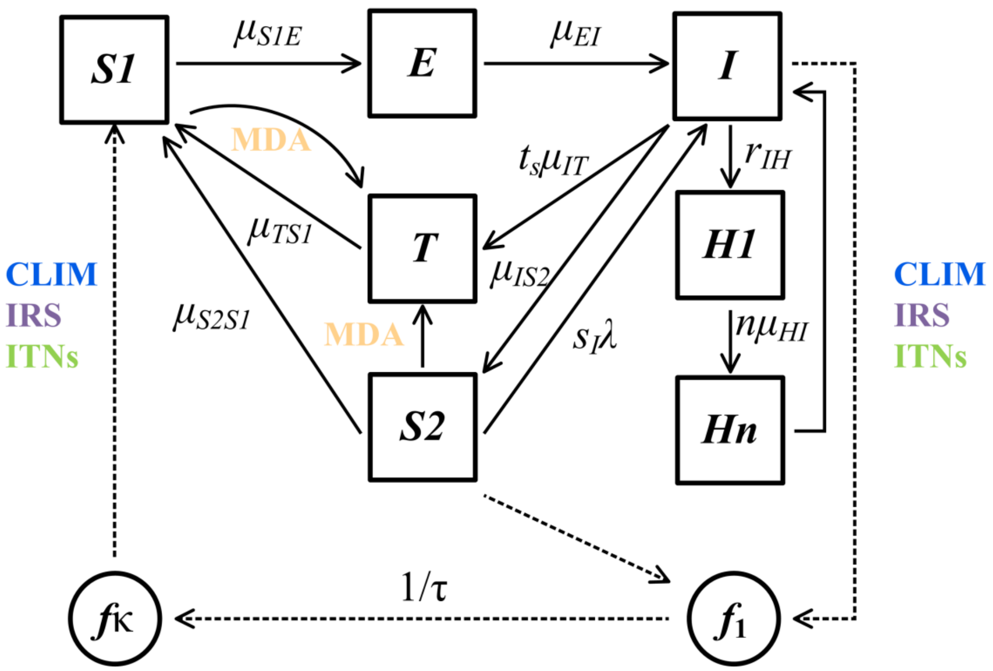

Fig. S4. Diagram of the model of malaria transmission. Human classes are represented as squares, and mosquito classes are represented as circles, with arrows indicating the rates of flow between classes. Human classes consist of *S*1 (susceptible), *E* (exposed), *I* (infected), *S*2 (recovered subpatent status, with partial immunity to reinfection), *H* (dormant liver stage, for the *P. vivax* model only), and *T* (antimalarial drug treatment with temporary immunity). The multiple *H* classes denote states where an individual is harbouring dormant hypnozoites in the liver. The mosquito classes included in the transmission model are represented as the force of infection resulting from infection in the human population during a previous period, *f*1, and the current rate of transmission experienced by a susceptible human, *f*κ. Dashed lines represent interactions between the human and mosquito. The role of mosquitoes in transmission is represented by a delayed equation between the latent *f* and current λ force of infection. CLIM, climate forcing.

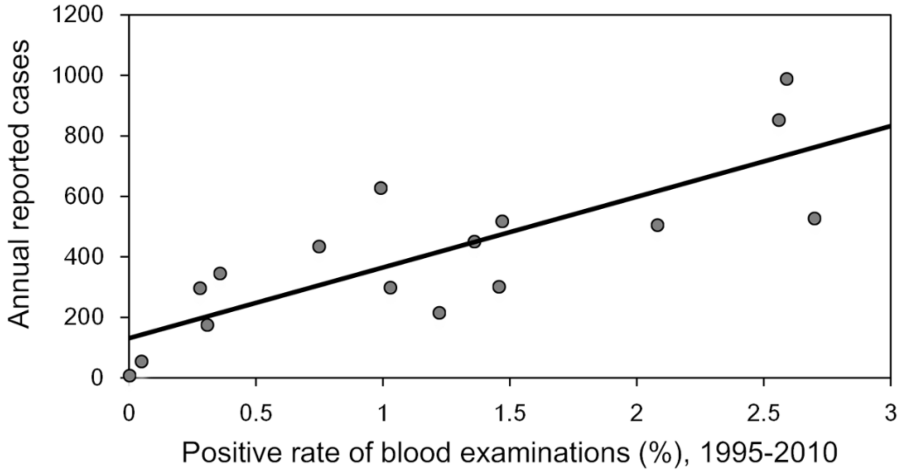

Fig. S5. Number of annual reported malaria cases and positive rate of bold examinations in study area, 1995–2010.

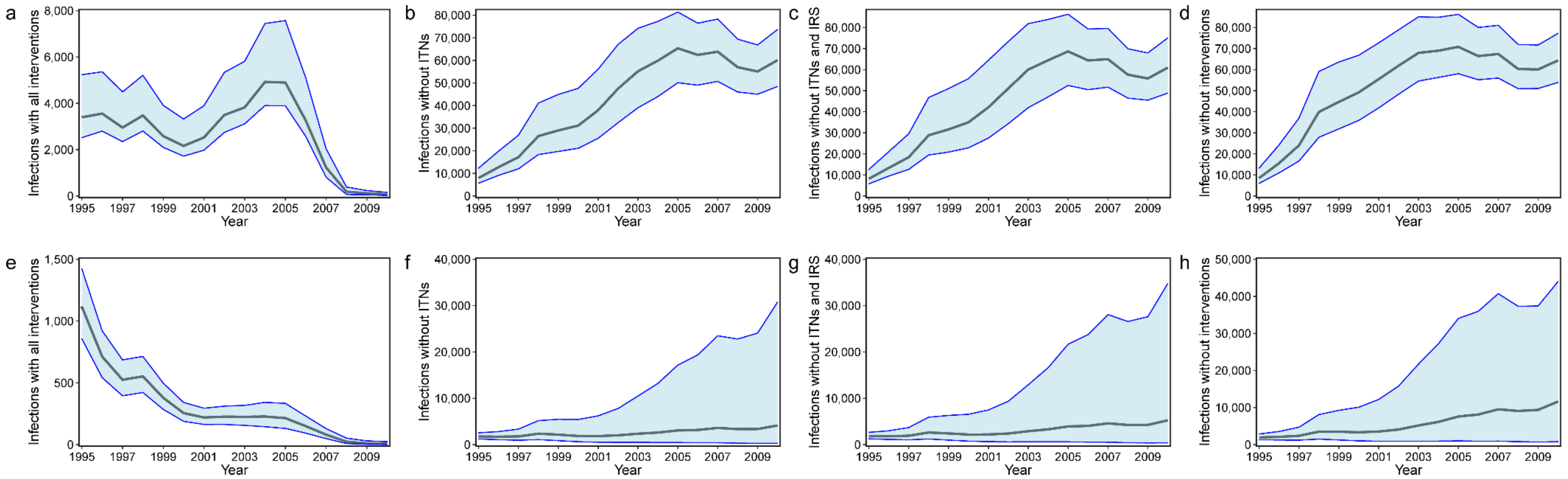

Fig. S6. Number of infections and 95% credible intervals with or without the interventions simulated by *P.vivax* model (a-d) and *P.falciparum* model (e-h).

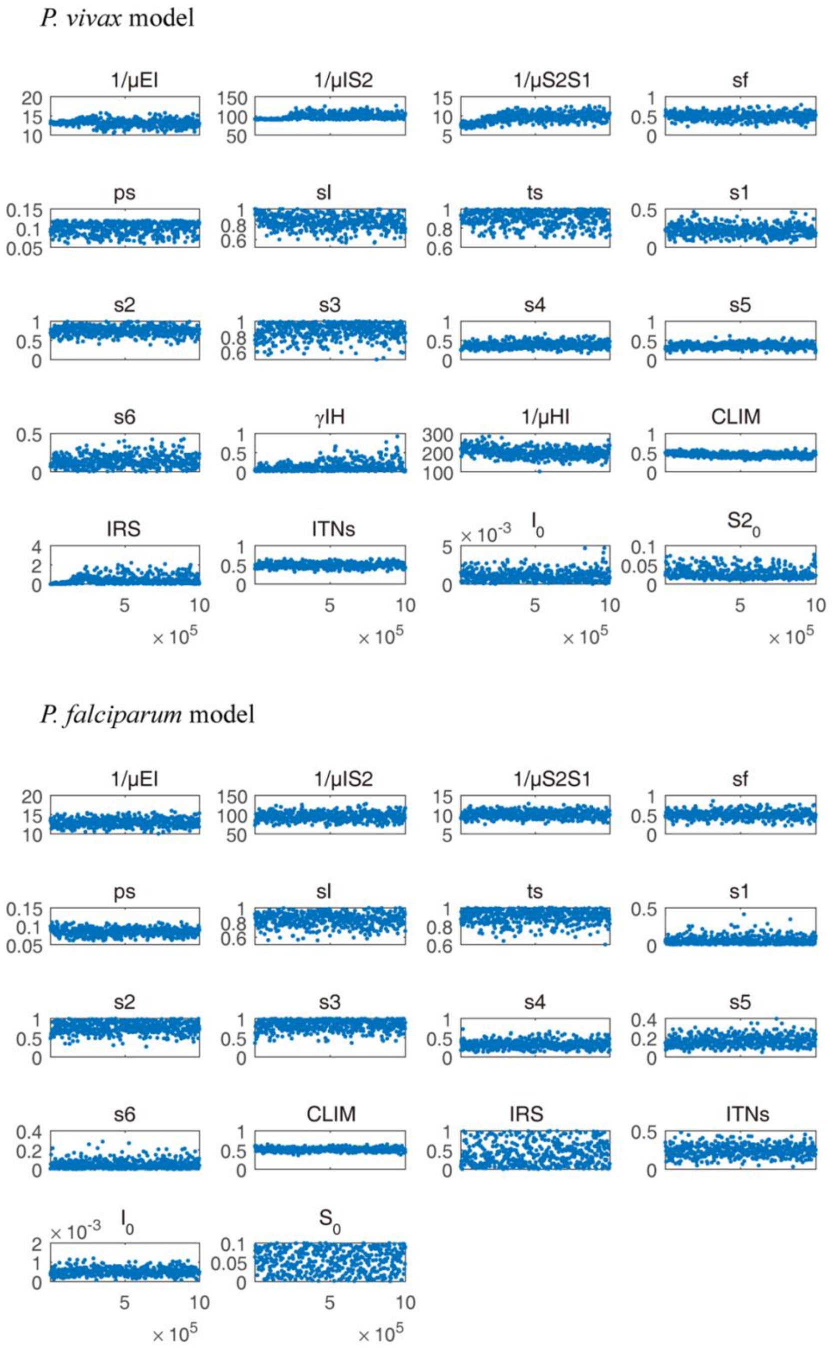

Fig. S7. Trace plots of the parameters for the *P. vivax* model (upper panels) and *P. falciparum* model (lower panels).

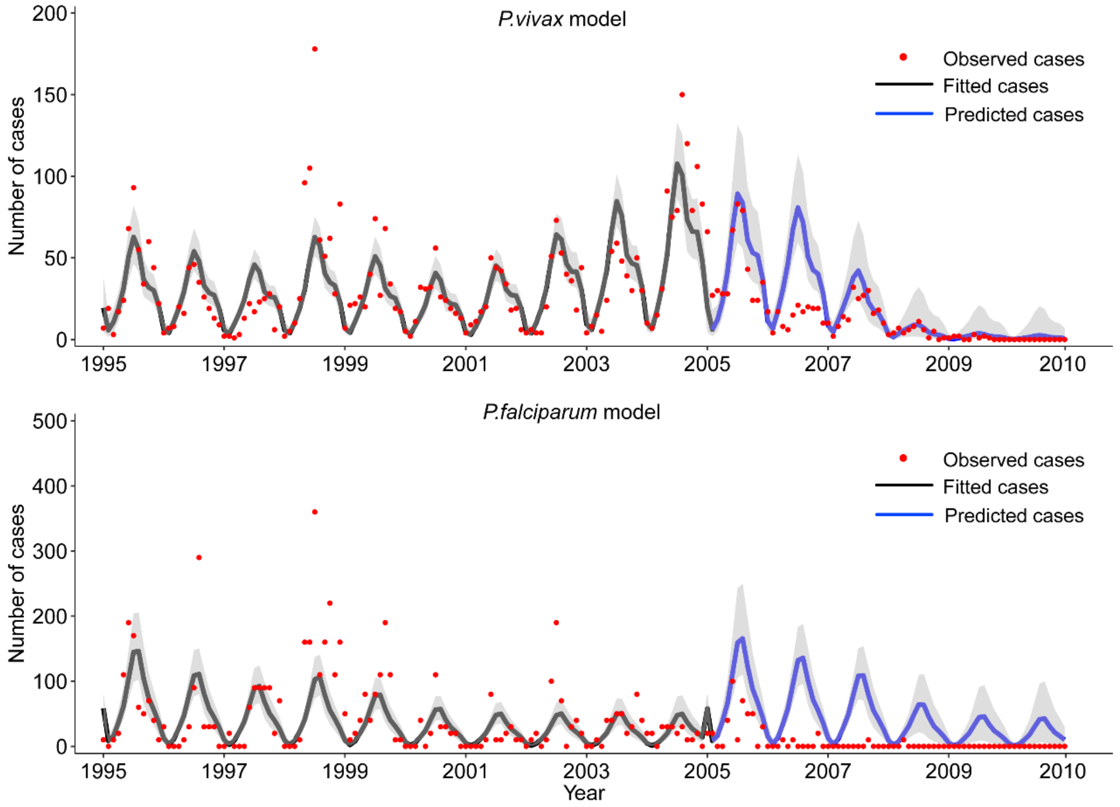

Fig. S8. Time series plot of observed and estimated malaria cases from 1995 to 2010 using epidemic model in our study. The models were fitted using 1995-2004 data and malaria cases were predicted using 2005-2010 data.

Table S1. Correlations between variables included in statistical and transmission model.

	**Pearson *r***	***P***
**MDA-ITNs**	0.46	0.08
**MDA-IRS**	0.06	0.83
**MDA-Niño 3.4**	-0.13	0.07
**ITNs-IRS**	-0.35	0.24
**ITNs-Niño 3.4**	-0.12	0.07
**IRS-Niño 3.4**	0.07	0.36

Table S2. Fitted parameters.

**Definition**	**Par.**	***PV***	***PF***	**Estimated**
**Reporting rate**	r	0.09 (0.02)	0.08 (0.01)	Y
**Time from exposure to infection**	$1/\mu_{EI}$	13 (1)	13 (1)	Y
**Time from infection to the subpatent status**	$1/\mu_{IS2}$	101 (7)	95 (10)	Y
**Time from the subpatent to susceptible status**	$1/\mu_{S2S1}$	10 (1)	10 (1)	Y
**Time to decreased immunity after treatment**	$1/\mu_{TS1}$	30	30	N
**Extrinsic incubation period**	τ	11	11	N
**Time of antimalarial treatment**	$1/\mu_{IT}$	8	8	N
**Treatment success**	t_S	0.89 (0.08)	0.90 (0.06)	Y
**Probability of relapse after unsuccessful treatment**	r_{IH}	0.22 (0.21)	—	Y
**Time from the dormant liver stage to infection**	$1/\mu_{HI}$	194 (25)	—	Y
**Superinfection from S2 to I**	s_I	0.83 (0.09)	0.83(0.08)	Y
**Infectivity of the S2 class**	s_f	0.52 (0.28)	0.49 (0.28)	Y
**Indoor residual spraying coefficient**	θ_{IRS}	0.51 (0.52)	0.39 (0.27)	Y
**Niño 3.4 coefficient**	θ_{CLIM}	0.42 (0.05)	0. 50 (0.04)	Y
**Insecticide-treated net coefficient**	θ_{ITNs}	0.48 (0.06)	0.23 (0.07)	Y
**Seasonality spline coefficient**	β_1	0.20 (0.06)	0.06 (0.05)	Y
	β_2	0.72 (0.12)	0.76 (0.13)	Y
	β_3	0.85 (0.11)	0.83 (0.13)	Y
	β_4	0.38 (0.08)	0.32 (0.09)	Y
	β_5	0.36 (0.07)	0.16 (0.05)	Y
	β_6	0.14 (0.08)	0.05 (0.05)	Y
**Initial condition *I* (=*N*×*I₀*)** → **Initial condition** I **(=**$N\times I_0$**)**	I_0	0.001 (0.006)	0.0005 (0.0001)	Y
**Initial condition** $S2$ **(=**$N\times S2_0$**)**	$S2_0$	0.02 (0.01)	0.05 (0.02)	Y

Table S3. Priors used in the epidemic model

Definition	Prior	Bounds
Reporting rate	0.08	0.06-0.12
Time from exposure to infection	13	10-20
Time from infection to the subpatent status	*PF*-100	60-200
	PV-115	90-200
Time from the subpatent to susceptible status	10	0-20
Treatment success	*PF*-0.9	0.7-1
	PV-0.9	0.7-1
Probability of relapse after unsuccessful treatment	*PV* -0.7	0-1
Time from the dormant liver stage to infection	*PV* -210	0-1000
Superinfection from S2 to I	0.5	0-1
Infectivity of the S2 class	0.5	0-1
Indoor residual spraying coefficient	0.01	0-Inf
Niño 3.4 coefficient	0.01	0-Inf
Insecticide-treated net coefficient	0.01	0-Inf
Seasonality spline coefficient	0.5	0-1
